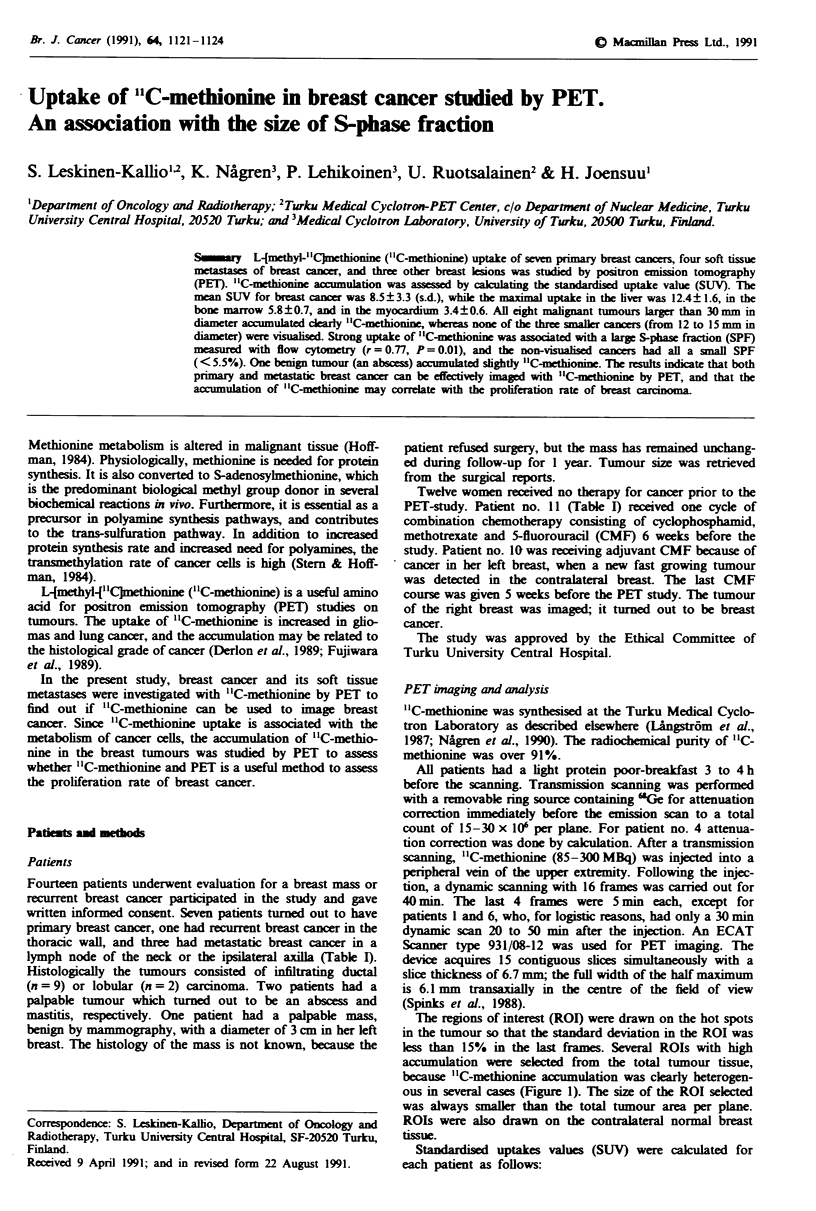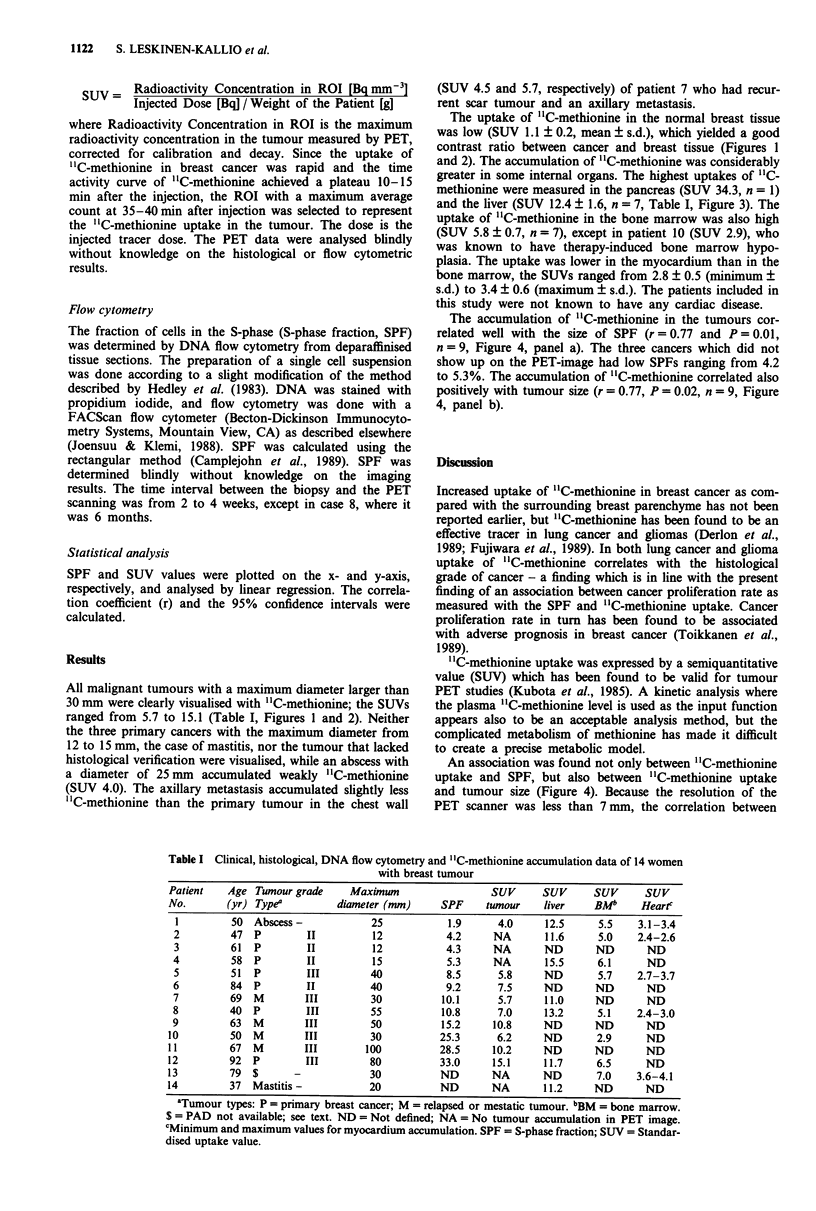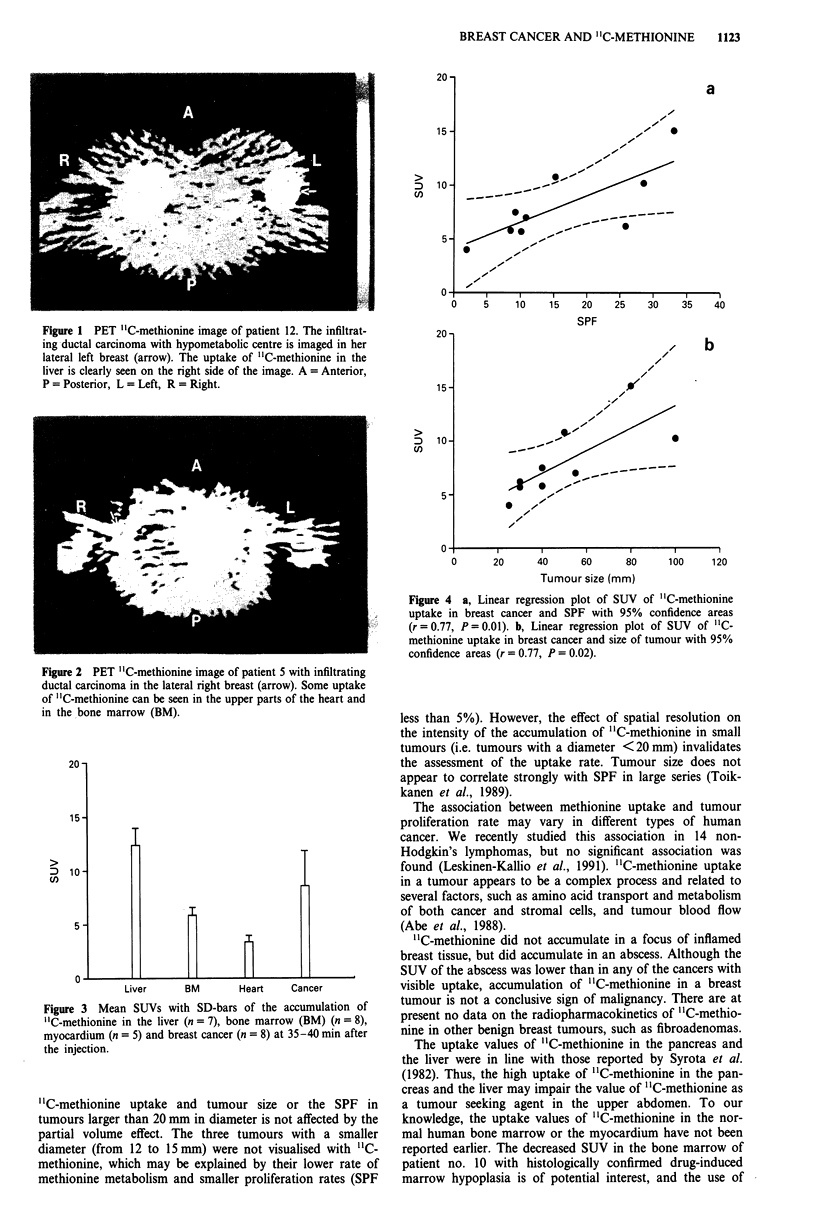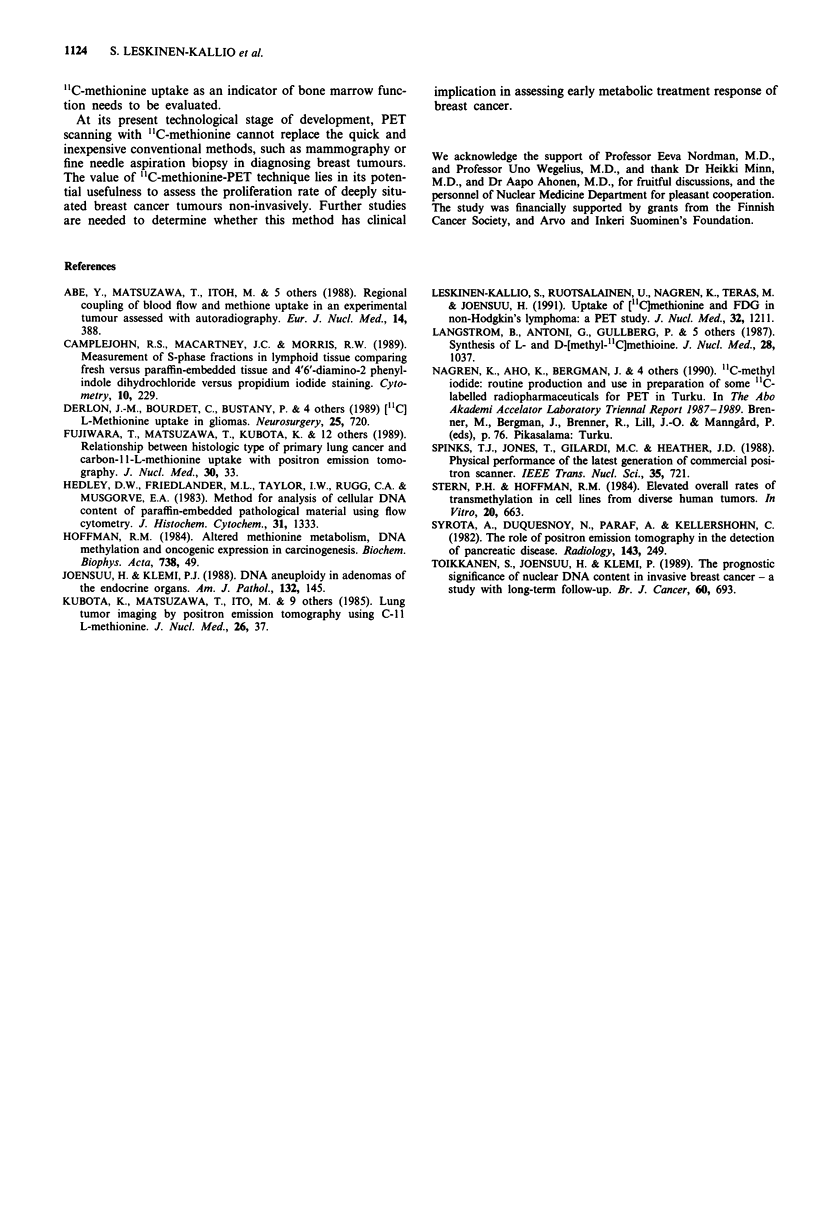# Uptake of 11C-methionine in breast cancer studied by PET. An association with the size of S-phase fraction.

**DOI:** 10.1038/bjc.1991.475

**Published:** 1991-12

**Authors:** S. Leskinen-Kallio, K. Någren, P. Lehikoinen, U. Ruotsalainen, H. Joensuu

**Affiliations:** Department of Oncology and Radiotherapy, Turku University Central Hospital, Finland.

## Abstract

**Images:**


Br. J. Cancer (1991), 64, 1121–1124 © Macmillan Press Ltd., 1991

### Uptake of $^{11}$C-methionine in breast cancer studied by PET. An association with the size of S-phase fraction

S. Leskinen-Kallio[1,2], K. Någren[3], P. Lehikoinen[3], U. Ruotsalainen[2] & H. Joensuu[1]

[1]*Department of Oncology and Radiotherapy;* [2]*Turku Medical Cyclotron-PET Center, c/o Department of Nuclear Medicine, Turku University Central Hospital, 20520 Turku; and* [3]*Medical Cyclotron Laboratory, University of Turku, 20500 Turku, Finland.*

**Summary** L-[methyl-$^{11}$C]methionine ($^{11}$C-methionine) uptake of seven primary breast cancers, four soft tissue metastases of breast cancer, and three other breast lesions was studied by positron emission tomography (PET). $^{11}$C-methionine accumulation was assessed by calculating the standardised uptake value (SUV). The mean SUV for breast cancer was 8.5 ± 3.3 (s.d.), while the maximal uptake in the liver was 12.4 ± 1.6, in the bone marrow 5.8 ± 0.7, and in the myocardium 3.4 ± 0.6. All eight malignant tumours larger than 30 mm in diameter accumulated clearly $^{11}$C-methionine, whereas none of the three smaller cancers (from 12 to 15 mm in diameter) were visualised. Strong uptake of $^{11}$C-methionine was associated with a large S-phase fraction (SPF) measured with flow cytometry ($r = 0.77$, $P = 0.01$), and the non-visualised cancers had all a small SPF (< 5.5%). One benign tumour (an abscess) accumulated slightly $^{11}$C-methionine. The results indicate that both primary and metastatic breast cancer can be effectively imaged with $^{11}$C-methionine by PET, and that the accumulation of $^{11}$C-methionine may correlate with the proliferation rate of breast carcinoma.

Methionine metabolism is altered in malignant tissue (Hoffman, 1984). Physiologically, methionine is needed for protein synthesis. It is also converted to S-adenosylmethionine, which is the predominant biological methyl group donor in several biochemical reactions *in vivo*. Furthermore, it is essential as a precursor in polyamine synthesis pathways, and contributes to the trans-sulfuration pathway. In addition to increased protein synthesis rate and increased need for polyamines, the transmethylation rate of cancer cells is high (Stern & Hoffman, 1984).

L-[methyl-[$^{11}$C]methionine ($^{11}$C-methionine) is a useful amino acid for positron emission tomography (PET) studies on tumours. The uptake of $^{11}$C-methionine is increased in gliomas and lung cancer, and the accumulation may be related to the histological grade of cancer (Derlon *et al.*, 1989; Fujiwara *et al.*, 1989).

In the present study, breast cancer and its soft tissue metastases were investigated with $^{11}$C-methionine by PET to find out if $^{11}$C-methionine can be used to image breast cancer. Since $^{11}$C-methionine uptake is associated with the metabolism of cancer cells, the accumulation of $^{11}$C-methionine in the breast tumours was studied by PET to assess whether $^{11}$C-methionine and PET is a useful method to assess the proliferation rate of breast cancer.

### Patients and methods

### *Patients*

Fourteen patients underwent evaluation for a breast mass or recurrent breast cancer participated in the study and gave written informed consent. Seven patients turned out to have primary breast cancer, one had recurrent breast cancer in the thoracic wall, and three had metastatic breast cancer in a lymph node of the neck or the ipsilateral axilla (Table I). Histologically the tumours consisted of infiltrating ductal ($n = 9$) or lobular ($n = 2$) carcinoma. Two patients had a palpable tumour which turned out to be an abscess and mastitis, respectively. One patient had a palpable mass, benign by mammography, with a diameter of 3 cm in her left breast. The histology of the mass is not known, because the patient refused surgery, but the mass has remained unchanged during follow-up for 1 year. Tumour size was retrieved from the surgical reports.

Twelve women received no therapy for cancer prior to the PET-study. Patient no. 11 (Table I) received one cycle of combination chemotherapy consisting of cyclophosphamid, methotrexate and 5-fluorouracil (CMF) 6 weeks before the study. Patient no. 10 was receiving adjuvant CMF because of cancer in her left breast, when a new fast growing tumour was detected in the contralateral breast. The last CMF course was given 5 weeks before the PET study. The tumour of the right breast was imaged; it turned out to be breast cancer.

The study was approved by the Ethical Committee of Turku University Central Hospital.

### *PET imaging and analysis*

$^{11}$C-methionine was synthesised at the Turku Medical Cyclotron Laboratory as described elsewhere (Långström *et al.*, 1987; Någren *et al.*, 1990). The radiochemical purity of $^{11}$C-methionine was over 91%.

All patients had a light protein poor-breakfast 3 to 4 h before the scanning. Transmission scanning was performed with a removable ring source containing $^{68}$Ge for attenuation correction immediately before the emission scan to a total count of 15–30 × $10^6$ per plane. For patient no. 4 attenuation correction was done by calculation. After a transmission scanning, $^{11}$C-methionine (85–300 MBq) was injected into a peripheral vein of the upper extremity. Following the injection, a dynamic scanning with 16 frames was carried out for 40 min. The last 4 frames were 5 min each, except for patients 1 and 6, who, for logistic reasons, had only a 30 min dynamic scan 20 to 50 min after the injection. An ECAT Scanner type 931/08-12 was used for PET imaging. The device acquires 15 contiguous slices simultaneously with a slice thickness of 6.7 mm; the full width of the half maximum is 6.1 mm transaxially in the centre of the field of view (Spinks *et al.*, 1988).

The regions of interest (ROI) were drawn on the hot spots in the tumour so that the standard deviation in the ROI was less than 15% in the last frames. Several ROIs with high accumulation were selected from the total tumour tissue, because $^{11}$C-methionine accumulation was clearly heterogenous in several cases (Figure 1). The size of the ROI selected was always smaller than the total tumour area per plane. ROIs were also drawn on the contralateral normal breast tissue.

Standardised uptakes values (SUV) were calculated for each patient as follows:

Correspondence: S. Leskinen-Kallio, Department of Oncology and Radiotherapy, Turku University Central Hospital, SF-20520 Turku, Finland.
Received 9 April 1991; and in revised form 22 August 1991.

$$SUV = \frac{\text{Radioactivity Concentration in ROI [Bq mm}^{-3}]}{\text{Injected Dose [Bq] / Weight of the Patient [g]}}$$

where Radioactivity Concentration in ROI is the maximum radioactivity concentration in the tumour measured by PET, corrected for calibration and decay. Since the uptake of $^{11}$C-methionine in breast cancer was rapid and the time activity curve of $^{11}$C-methionine achieved a plateau 10–15 min after the injection, the ROI with a maximum average count at 35–40 min after injection was selected to represent the $^{11}$C-methionine uptake in the tumour. The dose is the injected tracer dose. The PET data were analysed blindly without knowledge on the histological or flow cytometric results.

*Flow cytometry*

The fraction of cells in the S-phase (S-phase fraction, SPF) was determined by DNA flow cytometry from deparaffinised tissue sections. The preparation of a single cell suspension was done according to a slight modification of the method described by Hedley *et al.* (1983). DNA was stained with propidium iodide, and flow cytometry was done with a FACScan flow cytometer (Becton-Dickinson Immunocytometry Systems, Mountain View, CA) as described elsewhere (Joensuu & Klemi, 1988). SPF was calculated using the rectangular method (Camplejohn *et al.*, 1989). SPF was determined blindly without knowledge on the imaging results. The time interval between the biopsy and the PET scanning was from 2 to 4 weeks, except in case 8, where it was 6 months.

*Statistical analysis*

SPF and SUV values were plotted on the x- and y-axis, respectively, and analysed by linear regression. The correlation coefficient (r) and the 95% confidence intervals were calculated.

### Results

All malignant tumours with a maximum diameter larger than 30 mm were clearly visualised with $^{11}$C-methionine; the SUVs ranged from 5.7 to 15.1 (Table I, Figures 1 and 2). Neither the three primary cancers with the maximum diameter from 12 to 15 mm, the case of mastitis, nor the tumour that lacked histological verification were visualised, while an abscess with a diameter of 25 mm accumulated weakly $^{11}$C-methionine (SUV 4.0). The axillary metastasis accumulated slightly less $^{11}$C-methionine than the primary tumour in the chest wall (SUV 4.5 and 5.7, respectively) of patient 7 who had recurrent scar tumour and an axillary metastasis.

The uptake of $^{11}$C-methionine in the normal breast tissue was low (SUV 1.1 ± 0.2, mean ± s.d.), which yielded a good contrast ratio between cancer and breast tissue (Figures 1 and 2). The accumulation of $^{11}$C-methionine was considerably greater in some internal organs. The highest uptakes of $^{11}$C-methionine were measured in the pancreas (SUV 34.3, $n = 1$) and the liver (SUV 12.4 ± 1.6, $n = 7$, Table I, Figure 3). The uptake of $^{11}$C-methionine in the bone marrow was also high (SUV 5.8 ± 0.7, $n = 7$), except in patient 10 (SUV 2.9), who was known to have therapy-induced bone marrow hypoplasia. The uptake was lower in the myocardium than in the bone marrow, the SUVs ranged from 2.8 ± 0.5 (minimum ± s.d.) to 3.4 ± 0.6 (maximum ± s.d.). The patients included in this study were not known to have any cardiac disease.

The accumulation of $^{11}$C-methionine in the tumours correlated well with the size of SPF ($r = 0.77$ and $P = 0.01$, $n = 9$, Figure 4, panel a). The three cancers which did not show up on the PET-image had low SPFs ranging from 4.2 to 5.3%. The accumulation of $^{11}$C-methionine correlated also positively with tumour size ($r = 0.77$, $P = 0.02$, $n = 9$, Figure 4, panel b).

### Discussion

Increased uptake of $^{11}$C-methionine in breast cancer as compared with the surrounding breast parenchyme has not been reported earlier, but $^{11}$C-methionine has been found to be an effective tracer in lung cancer and gliomas (Derlon *et al.*, 1989; Fujiwara *et al.*, 1989). In both lung cancer and glioma uptake of $^{11}$C-methionine correlates with the histological grade of cancer – a finding which is in line with the present finding of an association between cancer proliferation rate as measured with the SPF and $^{11}$C-methionine uptake. Cancer proliferation rate in turn has been found to be associated with adverse prognosis in breast cancer (Toikkanen *et al.*, 1989).

$^{11}$C-methionine uptake was expressed by a semiquantitative value (SUV) which has been found to be valid for tumour PET studies (Kubota *et al.*, 1985). A kinetic analysis where the plasma $^{11}$C-methionine level is used as the input function appears also to be an acceptable analysis method, but the complicated metabolism of methionine has made it difficult to create a precise metabolic model.

An association was found not only between $^{11}$C-methionine uptake and SPF, but also between $^{11}$C-methionine uptake and tumour size (Figure 4). Because the resolution of the PET scanner was less than 7 mm, the correlation between

**Table I** Clinical, histological, DNA flow cytometry and $^{11}$C-methionine accumulation data of 14 women with breast tumour

| *Patient No.* | *Age (yr)* | *Tumour Type*[a] | *grade* | *Maximum diameter (mm)* | *SPF* | *SUV tumour* | *SUV liver* | *SUV BM*[b] | *SUV Heart*[c] |
|---|---|---|---|---|---|---|---|---|---|
| 1 | 50 | Abscess | – | 25 | 1.9 | 4.0 | 12.5 | 5.5 | 3.1–3.4 |
| 2 | 47 | P | II | 12 | 4.2 | NA | 11.6 | 5.0 | 2.4–2.6 |
| 3 | 61 | P | II | 12 | 4.3 | NA | ND | ND | ND |
| 4 | 58 | P | II | 15 | 5.3 | NA | 15.5 | 6.1 | ND |
| 5 | 51 | P | III | 40 | 8.5 | 5.8 | ND | 5.7 | 2.7–3.7 |
| 6 | 84 | P | II | 40 | 9.2 | 7.5 | ND | ND | ND |
| 7 | 69 | M | III | 30 | 10.1 | 5.7 | 11.0 | ND | ND |
| 8 | 40 | P | III | 55 | 10.8 | 7.0 | 13.2 | 5.1 | 2.4–3.0 |
| 9 | 63 | M | III | 50 | 15.2 | 10.8 | ND | ND | ND |
| 10 | 50 | M | III | 30 | 25.3 | 6.2 | ND | 2.9 | ND |
| 11 | 67 | M | III | 100 | 28.5 | 10.2 | ND | ND | ND |
| 12 | 92 | P | III | 80 | 33.0 | 15.1 | 11.7 | 6.5 | ND |
| 13 | 79 | $ | – | 30 | ND | NA | ND | 7.0 | 3.6–4.1 |
| 14 | 37 | Mastitis | – | 20 | ND | NA | 11.2 | ND | ND |

[a]Tumour types: P = primary breast cancer; M = relapsed or mestatic tumour. [b]BM = bone marrow. $ = PAD not available; see text. ND = Not defined; NA = No tumour accumulation in PET image. [c]Minimum and maximum values for myocardium accumulation. SPF = S-phase fraction; SUV = Standardised uptake value.

**Figure 1** PET $^{11}$C-methionine image of patient 12. The infiltrating ductal carcinoma with hypometabolic centre is imaged in her lateral left breast (arrow). The uptake of $^{11}$C-methionine in the liver is clearly seen on the right side of the image. A = Anterior, P = Posterior, L = Left, R = Right.

**Figure 2** PET $^{11}$C-methionine image of patient 5 with infiltrating ductal carcinoma in the lateral right breast (arrow). Some uptake of $^{11}$C-methionine can be seen in the upper parts of the heart and in the bone marrow (BM).

**Figure 3** Mean SUVs with SD-bars of the accumulation of $^{11}$C-methionine in the liver ($n = 7$), bone marrow (BM) ($n = 8$), myocardium ($n = 5$) and breast cancer ($n = 8$) at 35–40 min after the injection.

$^{11}$C-methionine uptake and tumour size or the SPF in tumours larger than 20 mm in diameter is not affected by the partial volume effect. The three tumours with a smaller diameter (from 12 to 15 mm) were not visualised with $^{11}$C-methionine, which may be explained by their lower rate of methionine metabolism and smaller proliferation rates (SPF less than 5%). However, the effect of spatial resolution on the intensity of the accumulation of $^{11}$C-methionine in small tumours (i.e. tumours with a diameter $<20$ mm) invalidates the assessment of the uptake rate. Tumour size does not appear to correlate strongly with SPF in large series (Toikkanen *et al.*, 1989).

**Figure 4** **a**, Linear regression plot of SUV of $^{11}$C-methionine uptake in breast cancer and SPF with 95% confidence areas ($r = 0.77$, $P = 0.01$). **b**, Linear regression plot of SUV of $^{11}$C-methionine uptake in breast cancer and size of tumour with 95% confidence areas ($r = 0.77$, $P = 0.02$).

The association between methionine uptake and tumour proliferation rate may vary in different types of human cancer. We recently studied this association in 14 non-Hodgkin's lymphomas, but no significant association was found (Leskinen-Kallio *et al.*, 1991). $^{11}$C-methionine uptake in a tumour appears to be a complex process and related to several factors, such as amino acid transport and metabolism of both cancer and stromal cells, and tumour blood flow (Abe *et al.*, 1988).

$^{11}$C-methionine did not accumulate in a focus of inflamed breast tissue, but did accumulate in an abscess. Although the SUV of the abscess was lower than in any of the cancers with visible uptake, accumulation of $^{11}$C-methionine in a breast tumour is not a conclusive sign of malignancy. There are at present no data on the radiopharmacokinetics of $^{11}$C-methionine in other benign breast tumours, such as fibroadenomas.

The uptake values of $^{11}$C-methionine in the pancreas and the liver were in line with those reported by Syrota *et al.* (1982). Thus, the high uptake of $^{11}$C-methionine in the pancreas and the liver may impair the value of $^{11}$C-methionine as a tumour seeking agent in the upper abdomen. To our knowledge, the uptake values of $^{11}$C-methionine in the normal human bone marrow or the myocardium have not been reported earlier. The decreased SUV in the bone marrow of patient no. 10 with histologically confirmed drug-induced marrow hypoplasia is of potential interest, and the use of

$^{11}$C-methionine uptake as an indicator of bone marrow function needs to be evaluated.

At its present technological stage of development, PET scanning with $^{11}$C-methionine cannot replace the quick and inexpensive conventional methods, such as mammography or fine needle aspiration biopsy in diagnosing breast tumours. The value of $^{11}$C-methionine-PET technique lies in its potential usefulness to assess the proliferation rate of deeply situated breast cancer tumours non-invasively. Further studies are needed to determine whether this method has clinical implication in assessing early metabolic treatment response of breast cancer.

We acknowledge the support of Professor Eeva Nordman, M.D., and Professor Uno Wegelius, M.D., and thank Dr Heikki Minn, M.D., and Dr Aapo Ahonen, M.D., for fruitful discussions, and the personnel of Nuclear Medicine Department for pleasant cooperation. The study was financially supported by grants from the Finnish Cancer Society, and Arvo and Inkeri Suominen's Foundation.

### References

ABE, Y., MATSUZAWA, T., ITOH, M. & 5 others (1988). Regional coupling of blood flow and methione uptake in an experimental tumour assessed with autoradiography. *Eur. J. Nucl. Med.*, **14**, 388.

CAMPLEJOHN, R.S., MACARTNEY, J.C. & MORRIS, R.W. (1989). Measurement of S-phase fractions in lymphoid tissue comparing fresh versus paraffin-embedded tissue and 4'6'-diamino-2 phenylindole dihydrochloride versus propidium iodide staining. *Cytometry*, **10**, 229.

DERLON, J.-M., BOURDET, C., BUSTANY, P. & 4 others (1989) [$^{11}$C] L-Methionine uptake in gliomas. *Neurosurgery*, **25**, 720.

FUJIWARA, T., MATSUZAWA, T., KUBOTA, K. & 12 others (1989). Relationship between histologic type of primary lung cancer and carbon-11-L-methionine uptake with positron emission tomography. *J. Nucl. Med.*, **30**, 33.

HEDLEY, D.W., FRIEDLANDER, M.L., TAYLOR, I.W., RUGG, C.A. & MUSGORVE, E.A. (1983). Method for analysis of cellular DNA content of paraffin-embedded pathological material using flow cytometry. *J. Histochem. Cytochem.*, **31**, 1333.

HOFFMAN, R.M. (1984). Altered methionine metabolism, DNA methylation and oncogenic expression in carcinogenesis. *Biochem. Biophys. Acta*, **738**, 49.

JOENSUU, H. & KLEMI, P.J. (1988). DNA aneuploidy in adenomas of the endocrine organs. *Am. J. Pathol.*, **132**, 145.

KUBOTA, K., MATSUZAWA, T., ITO, M. & 9 others (1985). Lung tumor imaging by positron emission tomography using C-11 L-methionine. *J. Nucl. Med.*, **26**, 37.

LESKINEN-KALLIO, S., RUOTSALAINEN, U., NAGREN, K., TERAS, M. & JOENSUU, H. (1991). Uptake of [$^{11}$C]methionine and FDG in non-Hodgkin's lymphoma: a PET study. *J. Nucl. Med.*, **32**, 1211.

LANGSTROM, B., ANTONI, G., GULLBERG, P. & 5 others (1987). Synthesis of L- and D-[methyl-$^{11}$C]methioine. *J. Nucl. Med.*, **28**, 1037.

NAGREN, K., AHO, K., BERGMAN, J. & 4 others (1990). $^{11}$C-methyl iodide: routine production and use in preparation of some $^{11}$C-labelled radiopharmaceuticals for PET in Turku. In *The Abo Akademi Accelator Laboratory Triennal Report 1987–1989*. Brenner, M., Bergman, J., Brenner, R., Lill, J.-O. & Manngård, P. (eds), p. 76. Pikasalama: Turku.

SPINKS, T.J., JONES, T., GILARDI, M.C. & HEATHER, J.D. (1988). Physical performance of the latest generation of commercial positron scanner. *IEEE Trans. Nucl. Sci.*, **35**, 721.

STERN, P.H. & HOFFMAN, R.M. (1984). Elevated overall rates of transmethylation in cell lines from diverse human tumors. *In Vitro*, **20**, 663.

SYROTA, A., DUQUESNOY, N., PARAF, A. & KELLERSHOHN, C. (1982). The role of positron emission tomography in the detection of pancreatic disease. *Radiology*, **143**, 249.

TOIKKANEN, S., JOENSUU, H. & KLEMI, P. (1989). The prognostic significance of nuclear DNA content in invasive breast cancer – a study with long-term follow-up. *Br. J. Cancer*, **60**, 693.